# RUNX2 promotes gastric cancer progression through the transcriptional activation of MGAT5 and MMP13

**DOI:** 10.3389/fonc.2023.1133476

**Published:** 2023-05-15

**Authors:** Ying Wang, Zhibo Tan, Xiaoyu Li, Lili Zhang, Xiaojuan Pei

**Affiliations:** ^1^ Department of Oncology, National Cancer Center/National Clinical Research Center for Cancer/Cancer Hospital and Shenzhen Hospital, Chinese Academy of Medical Science and Peking Union Medical College, Shenzhen, Guangdong, China; ^2^ Department of Radiation Oncology, Peking University Shenzhen Hospital, Shenzhen, Guangdong, China; ^3^ Department of Pathology, Shenzhen Hospital, Southern Medical University, Shenzhen, Guangdong, China

**Keywords:** cancer, gastric cancer, RUNX2, MGAT5, MMP13

## Abstract

**Introduction:**

RUNX2 is overexpressed in gastric cancer but the mechanism(s) through which it promotes tumor progression remain undefined. Here, we investigated the role of RUNX2 on gastric cancer pathogenesis at the molecular level.

**Methods:**

The qRT-PCR and western bolt were utilized to examine the mRNA and protein levels. CCK-8, Transwell and wound healing assays were used to measure cell proliferation, invasion and migration. CHIP-PCR gel electrophoresis was used to verify RUNX2 as a transcription factor for MMP13 and MGAT5. The *in vivo* assay was utilized to assess tumor growth. *In vivo* assay was used to evaluate tumor growth, aberrant expression of RUNX2 and lung metastasis of gastric cancer.

**Results:**

RUNX2 is overexpressed in MKN-45 and AGS cells. Genetic RUNX2 silencing reduced the proliferation, invasion and migration of MKN-45 and AGS cells. Analysis of the gastric cancer samples from the database revealed a significant positive correlation between MGAT5, MMP13, and RUNX2 expression. JASPAR analysis revealed that there was a potential binding site of RUNX2 in the promoter regions of MGAT5 and MMP13, and the experimental results confirmed that RUNX2 could regulate the expression of MGAT5 and MMP13 respectively. *In vivo* assays confirmed the aberrant expression of RUNX2 in mouse models of gastric cancer and reduced growth and lung metastasis in RUNX2 silenced xenograft tumors assessed.

**Conclusion:**

Collectively, these data reveal that RUNX2 enhances MGAT5 and MMP13 expression in gastric cancer cells and represents a biomarker and potential therapeutic target for gastric cancer therapy.

## Introduction

Gastric cancer is a leading cause of cancer-related death, with particularly high occurrence rates in East Asia. Gastric cancer patients typically present with advanced disease and accompanying lymph node metastasis, making treatment challenging (1, 2). The global incidence of gastric cancer diagnosed each year is increasing (1). In addition, despite considerable advances in chemotherapeutic interventions, the 5-year survival rate of gastric cancer patients is low and drug resistance is common (3). More effective biomarkers and therapeutic methods for gastric cancer therapy are urgently required.

Runt-associated transcription factor 2 (RUNX2) has been found to be overexpressed in gastric cancer and facilitate the invasive and metastatic properties of gastric cancer cells (4–8). RUNX2 has been proposed as a diagnostic biomarker for gastric cancer patients and is positively related to poor clinical prognosis and a range of clinicopathological characteristics (9). RUNX2 has been shown to enhance NID1 signaling, and promote malignant progression in gastric cancer by regulating COL1A1, YAP1 and FN1 expression (4, 8, 10). A systematic investigation of the role of RUNX during gastric cancer tumorigenesis has however not been performed.

MGAT5, also known as N-acetylglucosamino transferase V, is a N-glycan progression enzyme that translocates to the Golgi to catalyze the formation of β-1,6 branches of N-glycan (11). The number of MGAT5 glycan products typically increases in malignant tumors and is associated with disease progression (12, 13). MGAT5 deficiency inhibits the carcinogenic ability of polyomavirus intermediate T oncogene transgene in mice (14). MGAT5 has been shown to remodel the tumor microenvironment and accelerate tumor cell growth and metastasis through promoting the collapse of the extracellular matrix and enhancing the release of glycosyltransferase binding cytokines, including VEGF and MMPs (15). Hydrogen sulfide was shown to cause anti-gastric cancer effects through targeting MGAT5, highlighting its potential as a therapeutic target (16).

MMP13 is activated in gastric cancer and promotes the invasion of primary cancer cells (17). MMP13 has been reported as a downstream marker of RUNX2 in breast cancer cells (18). Therefore, we speculated that RUNX2 may also be the transcription factor for MGAT5. Hence, our experiments were conducted to investigate the relationships among RUNX2, MGAT5 and MMP13 in gastric cancer.

In this study, it was uncovered that RUNX2 activates the transcription of MGAT5 and MMP13 which act to facilitate gastric cancer metastasis *in vitro* and *in vivo*. This work may provide novel lights for the treatment of gastric cancer.

## Materials and methods

### Cell culture

Human gastric cancer cell lines MKN-45, AGS, and normal gastric mucosa epithelial cell line GES-1 were obtained from the Chinese Academy of Medical Sciences Shanghai Cell Bank (Shanghai, China). Cells were maintained in DMEM (Gibco Laboratories, USA) containing 10% fetal bovine serum (FBS, Gibco Laboratories, USA) and 1% penicillin-streptomycin in a 5% CO_2_ incubator at 37°C.

### Cell transfection

Si-negative control (si-NC) and si-RUNX2 were acquired from GenePharma Co. (Shanghai, China). These plasmids were transfected into MKN-45 and AGS cells through utilizing Lipofectamine 3000 reagents (Invitrogen, USA). After 48 hours, transfection efficiency was tested by qRT-PCR.

### qRT-PCR

Total RNA was extracted with TRIzol (Invitrogen) and cDNA was synthesized using a SuperScript™ III reverse transcriptase kit (Invitrogen, USA). qRT-PCRs were performed on an ABI Prism 7500 with SYBR Green qPCR Master Mix (Takara, Shanghai, China). qPCR conditions were as follows: 94°C for 30 s, followed by 40 cycles of 94°C for 5 s and 60°C for 30 s. The relative expression of RUNX2, MMP13 and MGAT5 were assayed using the 2^−ΔΔCt^ method. β-actin was used as an internal reference. Experiments were performed in triplicate. Primer sequences were as follows:

RUNX2, Forward: 5′-CCGGAATGCCTCTGCTGTTATGA-3′;

Reverse: 5′-ACTGAGGCGGTCAGAGAACAAACT-3′.

MMP13, Forward: 5′-TCCCTGCCCCTTCCCTATGG-3′;

Reverse: 5′-CTCGGAGCCTGTCAACTGTGG-3′.

MGAT5, Forward: 5′-AGGGCCATCCTGGGTTCATTA-3′;

Reverse: 5′-AAACTGCTGCGGGTTCAGATTC-3′.

β-actin, Forward: 5′-GGACATCCGCAAAGACCTGTA-3′;

Reverse: 5′-GCTCAGGAGGAGCAATGATCT-3′.

### Western blotting

Cells were lysed in RIPA buffer containing protease inhibitors. Proteins were resolved on 15% SDS-PAGE gels, transferred to PVDF membranes (Beyotime, Shanghai, China), and blocked. Membranes were probed with anti-RUNX2 (1 µg/ml; ab23981; Abcam), anti-MMP13 (1:5000; ab39012), anti-MGAT5 (1:1000; ab87977), anti-β-actin (1 µg/ml; ab8226) primary antibodies overnight at 4°C. Membranes were labeled with HRP conjugated secondary antibodies for 1 h at room temperature and bands were visualized using the ECL system (Amersham Biosciences, Inc). Band intensities were quantified using Quantity One software (BioRad).

### Cell counting kit-8 (CCK8) assays

MKN-45 and AGS cells were seeded into 96-well plates (1 000 cells/well) and assessed for viability using commercial CCK-8 kits (Dojindo Laboratories, Kumamoto, Japan). Briefly, cells were treated with 10 all CCK8 reagent for 1.5 h at 37°C and absorbance values read at 450 nm.

### Transwell assays

For invasion assays, MKN-45 and AGS cells were seeded into Transwell inserts pre-coated with Matrigel in serum-free medium (1 × 10^5^ cells/well). Media containing 10% FBS was added to the lower inserts for 24 h at 37˚C. Cells were fixed in 4% paraformaldehyde and stained with 0.1% crystal violet for 10 min. Invading cells were counted from five fields of view on a light microscope.

### Wound healing assays

Cell migration was determined using wound healing assays. Briefly, cells were seeded into 6-well plates (5 ×10^5^ cells/well) and grown to 100% confluency. A scratch was introduced using a 10 μL pipette tip and cells were incubated in serum-free medium for 24 h at 37°C in 5% CO_2_. Migrating cells were imaged on a light microscope.

### Enzyme-linked immunosorbent assay (ELISA)

MGAT5 expression was assayed using commercial ELISA plates (Abcam, Shanghai, China) coated with G-purified mouse monoclonal antibodies in BSA (2 μg/ml in coating buffer). Plates were blocked in serum and antibody labeled; Binding was detected with TMB plus stop solution. OD values were obtained at 450 nm.

### Bioinformatics analysis

The interaction between MGAT5, MMP13, and RUNX2 were analyzed using the Pearson correlation coefficient in gastric cancer cells using the GEPIA database. To further investigate the mechanism(s) through which RUNX2 modulates MGAT5 and MMP13, JASPAR (http://jaspar.genereg.net/) analysis was employed to identify putative promoter binding sites.

### Dual-luciferase reporter analysis

Cells were co-transfected with RUNX2 and PGL3-MMP13-3’UTR-WT, RUNX2 and PGL3-MMP13-3’UTR-mutant (mut), RUNX2 and PGL3-MGAT5-3’UTR-WT or RUNX2 and PGL3-MGAT5-3’UTR-mut using Lipofectamine 2000 (Invitrogen). Twenty-four hours post-transfection, cells were seeded into 96-well plates (1×10^4^/well) and luciferase activity was analyzed using the Dual-Luciferase Reporter Assay System.

### Chromatin immunoprecipitation (ChIP) assay

MKN-45 and AGS cells were cross-linked in 1% formaldehyde, nuclear proteins were extracted using EZ-Magna ChIP A/G Kits (Millipore). ChIP assays were performed as previously described (19). Data were quantified using ImageJ software.

### *In vivo* tumor formation assays

All assays were approved by our internal ethics committee. A total of 40 male BALB/c nude mice (5 weeks old, weight 18-22 g) were housed in a 30-60% humidity chamber at 20-25˚C under a 12:12 h light/dark cycle. Mice were provided food and water ad libitum. For establishment of the xenograft model, MKN-45 cells (1×10^7^) in 100 µl PBS were injected into mice at two sites. After 14 days, mice (total n=15) formed tumors and were divided into 3 groups (Control n=5; si-NC n=5, and si-RUNX2 n=5). For RUNX2 silencing, si-RUNX2 was injected into the tumor site. Tumor diameters were measured weekly (mm³) and tumor volumes calculated using the following equation: V=length × width^2^ ×1/2.

### Immunohistochemistry (IHC)

Sections were deparaffinized and probed with rabbit polyclonal anti-Ki-67 or rabbit polyclonal anti-RUNX2 primary antibodies at 4°C for 12 h. Sections were washed in TBST three times and incubated with HRP conjugated goat anti-mouse antibodies (#4414, Cell Signaling Technology). Sections were washed 3 times in TBST and signals were detected using the DAB Substrate kit. Images were captured on a light microscope (Leica).

### Hematoxylin-eosin (HE) staining

Lung sections were washed in PBS, fixed in 10% neutral buffered formalin (10 ml/FISH) at 4°C for 24 h, and dehydrated in 70% ethanol and xylene. Sections were paraffin-embedded and serially sectioned on a microtome (4 μm). Sections were stained with H&E and imaged on an optical microscope.

### Statistical analysis

Data are the mean ± standard deviation (SD). Experimental data were analyzed using SPSS 22.0 statistical and GraphPad Prism 8.0 software. Multi-group comparisons were performed using a Student’s t-test or One-way analysis of variance (ANOVA). Assays were repeated on a minimum of three occasions. P < 0.05 was deemed statistically significant.

## Results

### RUNX2 is highly expressed in gastric cancer cells

**Figure 1:**
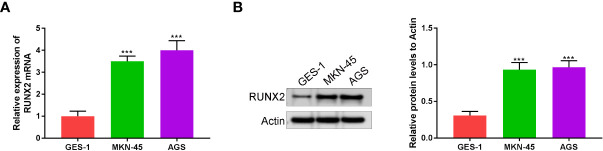
RUNX2 is highly expressed in gastric cancer cells. **(A)** RUNX2 mRNA expression level in MKN-45, AGS and GES-1 cells. **(B)** RUNX2 expression in MKN-45, AGS and GES-1 cell lines. ^***^p < 0.001 vs. GES-1.

RUNX2 expression in human gastric mucosal epithelial cell line (GES-1) and gastric cancer cell lines (MKN-45 and AGS) was investigated via qRT-PCR and western blotting. As shown in **Figures 1A, B** RUNX2 mRNA and protein levels were both highly expressed in MKN-45 and AGS cells, confirming that RUNX is upregulated in gastric cancer, consistent with previous studies.

### Knockdown of RUNX2 suppresses gastric cancer cell growth

**Figure 2:**
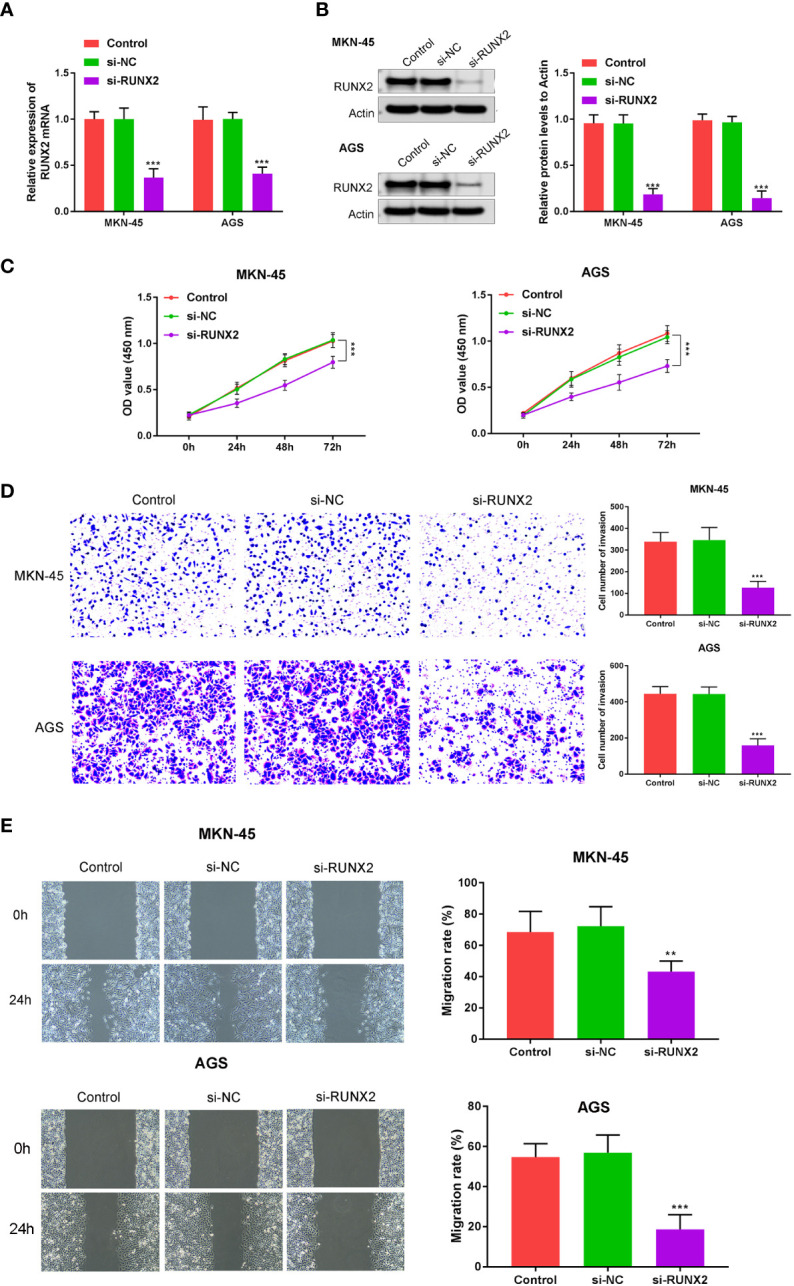
RUNX2 deficiency suppresses gastric cancer cell growth. **(A)** qRT-PCRs were performed to determine the mRNA expression of RUNX2 in MKN-45 and AGS cells. **(B)** Western blotting was performed to detect the expression of RUNX2 in MKN-45 and AGS cells. **(C)** Effects of RUNX2 overexpression assessed through CCK8 assays and **(D)** Transwell assays (bar = 100 µm). **(E)** Effects of RUNX2 silencing on MKN-45 and AGS cell migration determined through wound healing assays (bar = 100 µm). ^***^p < 0.001 vs. si-NC.

To explore the influences of RUNX2 on gastric cancer cell growth, invasion, and migration, qRT-PCR, western blotting, CCK-8, wound healing, and Transwell assays were performed in MKN-45 and AGS cells. Si-RUNX2 was transfected into MKN-45 and AGS cells, with si-NC transfected as a control. **Figures 2A, B** confirmed successful knockdown of RUNX2 was done in MKN-45 and AGS cells. CCK8 assays revealed the reduced viability of MKN-45 and AGS cells was found in si-RUNX2 group relative to si-NC or control groups, suggesting that the depletion of RUNX2 inhibited cell proliferation in MKN-45 and AGS cells (**Figure 2C**). Transwell and wound healing assays (**Figure 2D** **)** demonstrated that the down-regulation of RUNX2 also suppressed the invasion capacity of MKN-45 and AGS cells. The migration ability of MKN-45 and AGS cells were similarly inhibited after RUNX2 knockdown (**Figure 2E**). Collectively, these results demonstrated that the knockdown of RUNX2 suppressed the proliferation, invasion and migration abilities of gastric cancer cells.

### RUNX2 regulates the expression of MGAT5 and MMP13

**Figure 3:**
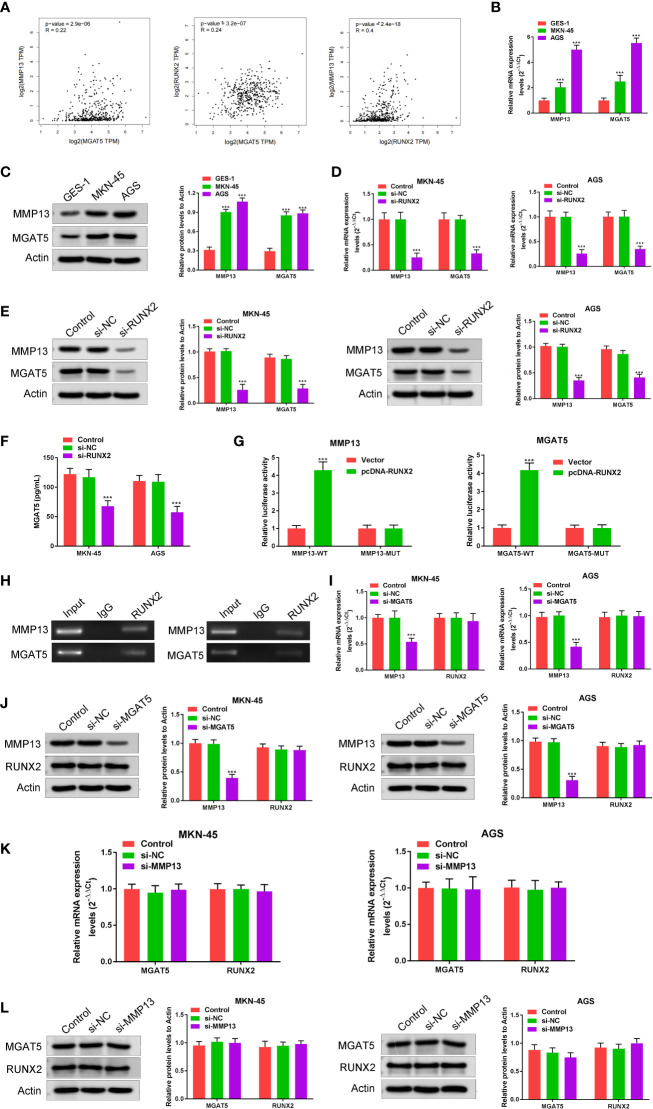
RUNX2 is a transcription factor that directly regulates the expression of MGAT5 and MMP13. **(A)** Correlation of MGAT5 and MMP13, MGAT5 and RUNX2, MMP13 and RUNX2 assayed through Pearson correlation coefficient using the GEPIA database. **(B)** qRT-PCRs were performed to determine the mRNA expression of MGAT5 and MMP13 in MKN-45 and AGS cells. ^***^p < 0.001 vs. GES-1. **(C)** Western blotting was utilized to assay MGAT5 and MMP13 expression in MKN-45 and AGS cells. ^***^p < 0.001 vs. GES-1. **(D)** qRT-PCRs analysis. ^***^p < 0.001 vs. si-NC. **(E)** Western blotting for MGAT5 and MMP13 in RUNX2 silenced MKN-45 and AGS cells. ^***^p < 0.001 vs. si-NC. **(F)** ELISA to assay the impact of RUNX2 knockout on MGAT5 expression. ^***^p < 0.001 vs. si-NC. **(G)** Dual luciferase in cells transfected with WT, MUT 3’-UTR of MMP13 or MGAT5. ^***^p < 0.001 vs. vector. **(H)** CHIP-PCR gel electrophoresis to verify RUNX2 as a transcription factor for MMP13 and MGAT5. **(I)** qRT-PCR in MGAT5 silenced cells. ^***^p < 0.001 vs. si-NC. **(J)** Western blotting of RUNX2 and MMP13 expression in MGAT5 silenced cells. ^***^p < 0.001 vs. si-NC. **(K)** qRT-PCR analysis of MGAT5 and RUNX2 in MMP13 silenced cells. **(L)** Western blotting of MGAT5 and RUNX2 in MMP13 silenced MKN-45 and AGS cells.

To explore the relationship among RUNX2, MGAT5, and MMP13 in gastric cancer cells, GEPIA and Jaspar databases were explored. As shown in **Figure 3A**, MGAT5, MMP13, and RUNX2 were significantly positively associated with each other in gastric cancer based on the GEPIA database. The Jaspar database indicated that RUNX2 is a transcription factor for MMP13 and revealed five potential RUNX2 binding sites within the MMP13 promoter (**Table 1**). Moreover, the Jaspar database showed that RUNX2 is a transcription factor of MGAT5 (gene bank name AK125207) identifying six potential RUNX2 binding sites (**Table 2**). The qRT-PCR and western blotting showed higher expression of MGAT5 and MMP13 mRNA and protein were discovered in MKN-45 and AGS cells (**Figures 3B, C**). The si-RUNX2 group showed lower MGAT5 and MMP13 mRNA and protein expression relative to si-NC or control groups (**Figures 3D, E**). The reduction in MGAT5 expression following RUNX2 silencing was further confirmed by ELISA (**Figure 3F**). RUNX overexpression also increased the expression of luciferase reporter constructs of MMP13-WT or MGAT5-WT promoter regions, but had no effect on MMP13-MUT or MGAT5-MUT lacking the putative RUNX2 binding sites (**Figure 3G**). ChIP assay further confirmed the association of RUNX2 with the MMP13 and MGAT5 promoter regions (**Figure 3H**). MGAT5 silencing had no effects on RUNX2 expression in MKN-45 and AGS cells, but decreased MMP13 expression at both the mRNA and protein level (**Figures 3I, J**). MMP13 silencing had no effect on either RUNX2 or MGAT5 (**Figures 3K, L**). Together, these data revealed a role for RUNX2 in independently regulating the expression of MGAT5 and MMP13, the RUNX2/MGAT5/MMP13 positive axis in gastric cancer.

**Table 1:** The binding site of Runx2 and MMP13.

Matrix ID	Name	Score	Relative score	Start	End	Strand	Predicted sequence
MA0511.2	MA0511.2.RUNX2	11.24984	0.932235	1458	1466	–	CAACCGCAG
MA0511.2	MA0511.2.RUNX2	11.04085	0.928382	1861	1869	+	AAACCACAC
MA0511.2	MA0511.2.RUNX2	8.370338	0.879158	1854	1862	+	CTACCACAA
MA0511.1	MA0511.1.RUNX2	11.34579	0.874991	1853	1867	–	GTGGTTTGTGGTAGA
MA0511.1	MA0511.1.RUNX2	9.841478	0.85178	1860	1874	–	CCCGAGTGTGGTTTG

**Table 2:** The binding site of RUNX2 and MGAT5.

Matrix ID	Name	Score	Relative score	Start	End	Strand	Predicted sequence
MA0511.2	MA0511.2.RUNX2	11.76725	0.941772	410	418	–	aaaccacag
MA0511.1	MA0511.1.RUNX2	14.98088	0.931077	405	419	+	tttccctgtggtttt
MA0511.2	MA0511.2.RUNX2	9.341541	0.89706	1419	1427	+	agaccacag
MA0511.1	MA0511.1.RUNX2	12.13177	0.887118	1418	1432	–	ggagactgtggtctc
MA0511.2	MA0511.2.RUNX2	7.435408	0.861924	620	628	+	agacctcaa
MA0511.2	MA0511.2.RUNX2	6.809718	0.850391	168	176	+	taaccaccc

### Effects on RUNX2 on gastric cancer cells are mediated through MMP13 and MGAT5

**Figure 4:**
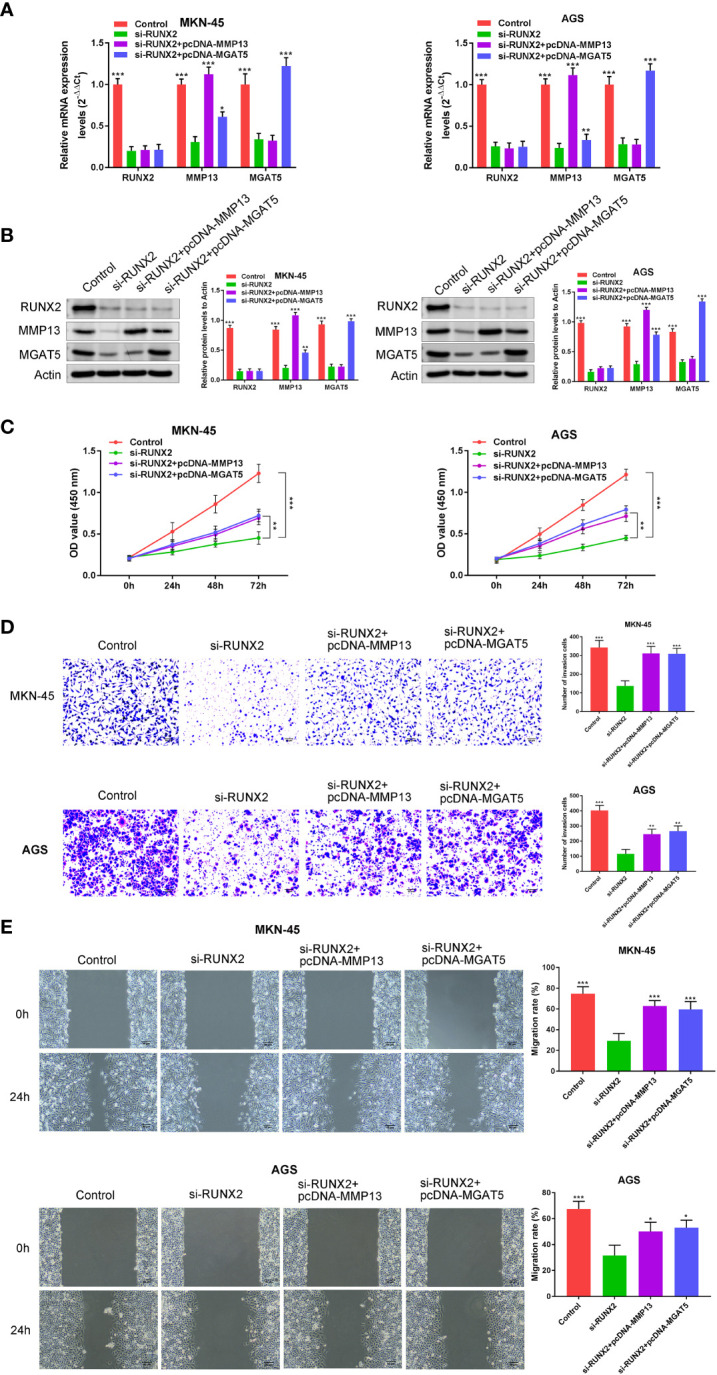
MMP13 and MGAT5 mediate the regulatory process of RUNX2 on gastric cancer progression. **(A)** qRT-PCR to assay the expression of RUNX2, MMP13 and MGAT5 in MKN-45 and AGS cells. **(B)** Western blotting is showing the protein expression levels of RUNX2, MMP13 and MGAT5 in MKN-45 and AGS cells. **(C)** MKN-45 and AGS cell growth was analyzed by CCK8 assay. **(D)** Cell invasion were tested by transwell assay (bar = 100 µm). **(E)** Cell migration were detected by wound healing assay (bar = 100 µm). *p<0.05, ^**^p < 0.01, ^***^p < 0.001 vs. si-RUNX2.

We next examined whether the effects of RUNX2 on gastric cancer cells were mediated by the overexpression of MMP13 and MGAT5. MKN-45 and AGS cells were simultaneously transfected with si-RUNX2 and pcDNA-MMP13 or pcDNA-MGAT5 plasmids. Expression of the proteins was confirmed by both western blotting and RT-PCR analysis, it was demonstrated that compared with the Control group, RUNX2 expression was all down-regulated in the si-RUNX2, si-RUNX2+pcDNA-MMP13 or si-RUNX2+pcDNA-MGAT5 group; MMP13 expression was down-regulated in the si-RUNX2 group, but this change was reversed in the si-RUNX2+pcDNA-MMP13 or si-RUNX2+pcDNA-MGAT5 group; MGAT5 expression was down-regulated in the si-RUNX2 or si-RUNX2+pcDNA-MMP13 group, but this change was reversed in the si-RUNX2+pcDNA-MGAT5 group (**Figures 4A, B**). CCK-8 assays were used to detect changes in cell viability after simultaneous transfection of the constructs. **Figure 4C** shows that the decreased cell proliferation observed following RUNX2 silencing could be reversed by the overexpression of MMP13 or MGAT5. Similarly, Transwell assays uncovered that the reduction of cell invasion and migration following RUNX2 knockdown were rescued by MMP13 or MGAT5 overexpression (**Figures 4D, E**). These data demonstrate that MMP13 and MGAT5 mediated the regulatory processes of RUNX2 on gastric cancer progression.

### *In vivo* effects of RUNX2 ablation on cancer growth

**Figure 5:**
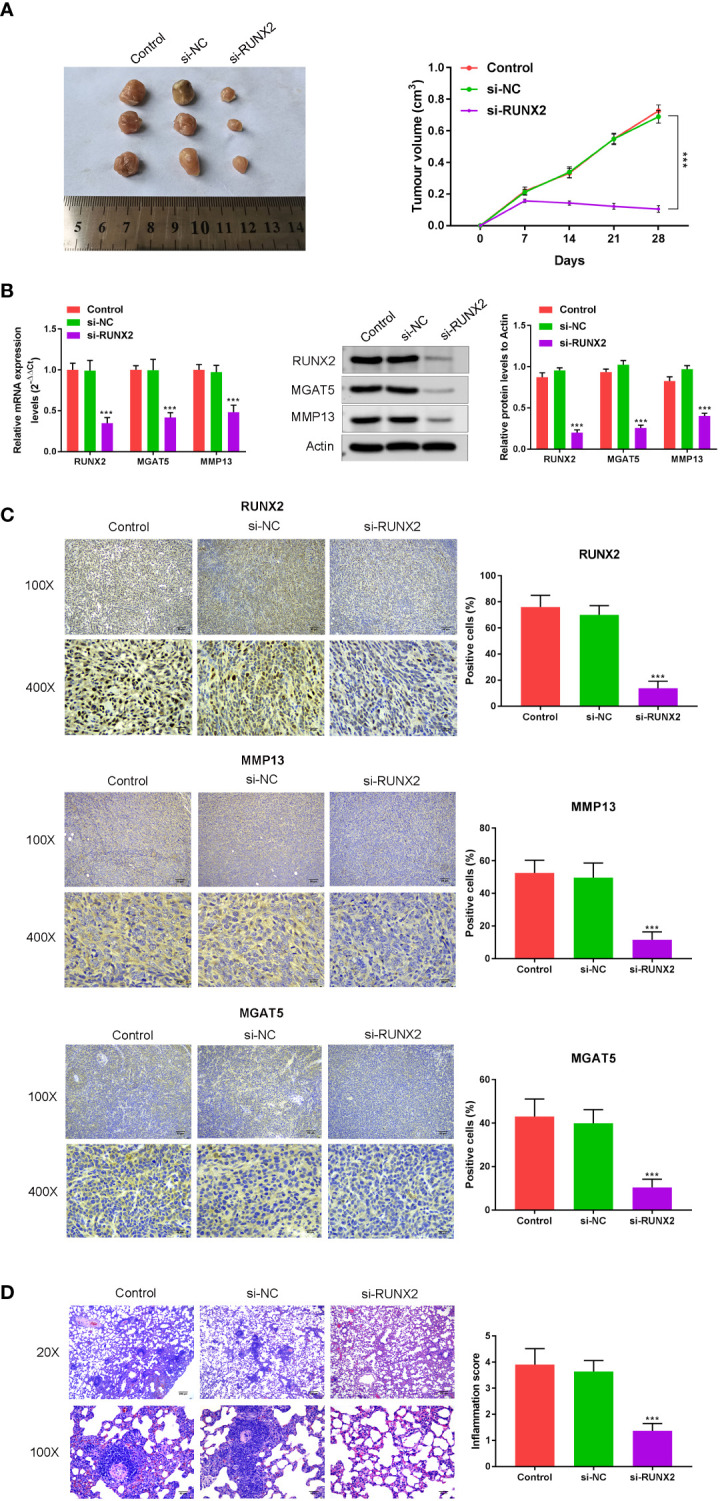
*In vivo* confirmation of the influence of RUNX2 deficiency on cancer growth and MGAT5 and MMP13 expression. Mice (total n=15) were divided into 3 groups (n=5 per-group; Control, si-NC, and si-RUNX2) randomly. **(A)** Tumors were collected from mice and Tumor volumes. **(B)** qRT-PCR and Western blotting were used to assay the expression of RUNX2, MMP13 and MGAT5. **(C)** Immunohistochemical analysis of RUNX2, MMP13 and MGAT5 levels, and the positive cells (%) of RUNX2, MMP13 and MGAT5 were confirmed (bar graph). **(D)** H&E was used to verify that the influence of RUNX2 on lung metastasis of gastric cancer. ^***^p < 0.001 vs. si-NC.

To understand the influence of RUNX2 knockdown on tumor growth and MMP13/MGAT5 expression *in vivo*, subcutaneous xenograft tumor models were generated in male Balb/c nude mice (n=5 mice in each group). In mice subcutaneously injected with MKN-45 cells (Control, si-NC and si-RUNX2), both tumor size and volumes were decreased following RUNX2 silencing (**Figure 5A**). RUNX2 knockdown also led to the decreased levels of RUNX2, MMP13 and MGAT5 in mice tumor tissues when assessed by western blotting or IHC assay (**Figures 5B, C**). **Figure 5D** showed that RUNX2 silencing also decreased cancer metastasis with fewer lung nodules following H & E staining. Together, these findings confirmed that RUNX2 knockdown inhibited gastric cancer tumor growth in mice.

## Discussion

Despite of considerable advances in the detection and treatment of gastric cancer, the underlying mechanism(s) mediating its progression remain largely undefined. Previous studies have shown that RUNX2 is overexpressed in gastric cancer cells and acts to promote their invasion and metastatic capacity (7). Here, we elucidated the role of RUNX2 in the tumorigenesis of gastric cancer cells using the GEPIA database. This revealed a significant positive correlation between MGAT5, MMP13 and RUNX2 expression. JASPAR analysis revealed novel RUNX2 binding sites in the MGAT5 and MMP13 promoters, which we confirmed through dual luciferase reporter and CHIP assays. The role of the RUNX2-MGAT5-MMP13 in the self-renewal and tumorigenesis of gastric cancer cell was then examined both *in vitro* and *in vivo*. We found that RUNX2 is highly expressed in gastric cancer cell lines, the silencing of which impaired gastric cancer growth and metastasis. We further found that the effects of RUNX2 are mediated through the activation of MGAT5 and MMP13 both *in vitro* and *in vivo*, as their overexpression could compensate for RUNX2 depletion in gastric cancer cell lines and xenograft tumor models. Collectively, these data reveal RUNX2-MGAT5-MMP13 as key biomarkers and novel therapeutic targets for much needed anti-cancer therapeutics.

RUNX2 is best characterized as an essential factor during osteoblast differentiation and bone development, but possesses a wide tissue distribution (20). RUNX2 has been shown to participate in the regulation of mammary specific gene expression and metastatic cancer progression in established breast cell lines (21). In breast cancer, RUNX2 expression correlates with the triple negative subtype through cross-talk with estrogen receptor signaling (22–24). RUNX2 also interacts with a number of cell cycle regulators, including cyclin-dependent kinases, pRB and p21Cip1 in addition to p53 (25). It has been showed that ABL phosphorylates and activates RUNX2 through its SH2 domain and that the formation of the ABL-RUNX2 complex enhances the expression of MMP13 (26, 27). Previous studies have highlighted COL1A1, YAP1 and chemokine receptor CXCR4 as targets of RUNX2 that facilitate the *in vitro* invasion and metastatic potential of gastric cancer cells (4, 7). Elevated RUNX2 expression has also been reported as a prognostic marker of poor clinical outcome and a promising molecular target for the treatment of the patients with pancreatic cancer (4). Consistent with previous studies, we observed high RUNX2 expression in gastric cancer cells which promoted MMP13 and MGAT5 expression. MMP13 or (Collagenase-3) and MGAT5 play known roles in tumor metastasis and invasion in gastrointestinal tract tumors, head and neck squamous carcinomas esophageal cancer and colorectal tumors. MMP13 and MGAT5 have been investigated in gastric cancer. Hydrogen sulfide inhibits MGAT5 and displays antitumor efficacy in gastric cancer (16) MMP13 has been shown to be upregulated in gastric cancer (17) and can be inhibited by SIRT1 which modulates the STAT3/MMP13 axis to suppress cell proliferation and metastasis in gastric cancer (28). GOLM1 also upregulates MMP13 to promote the invasiveness of gastric cancer cells (29).

In summary, we identify MGAT5 and MMP13 as novel RUNX2 targets with known metastatic potential in gastric cancer cells and tissue. This highlights RUNX2, MGAT5 and MMP13 as key biomarkers of metastatic gastric cancer and therapeutic targets for much-needed anti-gastric cancer drugs.

## Data availability statement

The original contributions presented in the study are included in the article/supplementary material. Further inquiries can be directed to the corresponding author.

## Author contributions

YW and XP initiated the project, designed the experiments, wrote the manuscript, and interpreted the data. XL and LZ performed the experiments. ZT and XL finished the data analysis. All authors contributed to the article and approved the submitted version.
